# Machine Learning Calibration of Smartphone-Based Infrared Thermal Cameras: Improved Bias and Persistent Random Error

**DOI:** 10.3390/s26041295

**Published:** 2026-02-17

**Authors:** Jayroop Ramesh, Tom Loney, Stefan Du Plessis, Homero Rivas, Assim Sagahyroon, Fadi Aloul, Thomas Boillat

**Affiliations:** 1 Department of Computer Science and Engineering, American University of Sharjah, Sharjah 26666, United Arab Emirates; b00057412@aus.edu (J.R.); faloul@aus.edu (F.A.); 2 College of Medicine, Mohammed Bin Rashid University of Medicine and Health Sciences, Dubai 505055, United Arab Emirates; tom.loney@dubaihealth.ae (T.L.);

**Keywords:** thermal images, thermography, smartphone plug-in cameras, bland-altman, skin temperature, machine learning, calibration

## Abstract

Low-cost, smartphone-based thermal cameras offer unprecedented accessibility for physiological monitoring, yet their validity and reliability for absolute skin temperature measurement in clinical settings remain contentious. This study aims to quantify the agreement and repeatability of a widely used smartphone thermal camera, the FLIR One Pro, against a consumer-grade, non-contact infrared thermometer, the iHealth PT3. A method comparison study was conducted with 40 healthy adult participants, yielding a total of 2400 temperature measurements. Skin temperature of the hand dorsum was measured concurrently with the FLIR One Pro and the iHealth PT3. The protocol involved two rounds: Round 1 (R1) in a stable, static environment to assess baseline repeatability, and Round 2 (R2) in a dynamic environment mimicking clinical repositioning. The performance of the instruments was compared using paired *t*-tests for mean differences and Bland–Altman analysis for assessing agreement. The iHealth PT3 demonstrated superior precision, with an average intra-participant standard deviation (SD) of 0.030 °C in R1 and 0.092 °C in R2. In stark contrast, the FLIR One Pro exhibited significantly higher variability, with an average SD of 0.34 °C in R1 and 0.30 °C in R2. Bland–Altman analysis revealed a substantial mean bias of −1.42 °C in R1 and −1.15 °C, with critically wide 95% limits of agreement ranges of ≈6 °C. The substantial systematic bias and poor agreement of the FLIR One Pro far exceed both its manufacturer-stated accuracy and clinically acceptable error margins for absolute temperature measurement. To further examine whether calibration could mitigate these deficiencies, we applied a suite of ten machine learning regressors to map FLIR readings onto iHealth PT3 values. Calibration reduced systematic bias across all models, with Quantile Gradient-Boosted Regression Trees achieving the lowest MAE (1.162 °C). The Extra Trees model yielded the lowest RMSE (1.792 °C) and the highest explained variance ($R^{2}$ = 0.152), yet this relatively low value confirms that the device’s high intrinsic variability limits the effectiveness of algorithmic correction. As such the device has limited utility for longitudinal patient monitoring or for diagnostic decisions that rely on precise, absolute temperature thresholds. These findings inform medical practitioners in low-resource settings of the profound limitations of using this device as a standalone clinical thermometer and emphasize that algorithmic correction cannot compensate for fundamental hardware and measurement noise constraints.

## 1. Introduction

The association between body temperature and disease is a foundational principle of medicine, recognized since antiquity. Body temperature stands as a cardinal vital sign, with even minor deviations from the narrow range of normothermia serving as critical indicators of underlying physiological dysfunction [1]. Fluctuations in core or peripheral temperature can signal a vast array of conditions, including systemic infection, localized inflammation, metabolic dysregulation, and vascular disorders [2]. The ability to measure temperature accurately and reliably is therefore not merely a procedural task but a cornerstone of clinical diagnosis, patient monitoring, and therapeutic evaluation. For centuries, this has been the domain of contact thermometers, which have evolved but remain limited to providing single-point measurements.

A significant paradigm shift in temperature measurement occurred with the application of infrared (IR) physics to medicine. This was predicated on the discovery that human skin behaves as a near-perfect blackbody radiator, emitting thermal energy in the infrared spectrum in direct proportion to its surface temperature [3]. This principle gave rise to infrared thermography, a technology that captures this emitted radiation to create a visual map, or thermogram, of the body’s surface temperature distribution [1]. The primary advantages of this modality are profound: it is entirely non-invasive, contactless (thereby eliminating risks of cross-contamination), and provides a rich, spatial representation of thermal patterns rather than an isolated point measurement [4]. These characteristics have made it an invaluable tool in a diverse range of medical applications, from the assessment of vascular integrity in diabetic neuropathy and the monitoring of inflammatory activity in rheumatic diseases to mass fever screening during public health crises and the evaluation of burn wound severity [5].

The historical trajectory of thermal imaging technology has been one of progressive miniaturization and democratization. Early thermal cameras were cumbersome, requiring complex setups and liquid nitrogen cooling, which restricted their use to highly specialized research settings [1]. The development of uncooled focal plane array detectors in the late 20th century marked a critical turning point, leading to smaller, more portable, and user-friendly devices that could be operated by non-specialized staff in various clinical environments [6]. The most recent and disruptive evolution in this field has been the advent of ultra-portable, plug-in thermal cameras designed to interface directly with smartphones [7].

Devices such as the FLIR One Pro leverage the sophisticated computing power, high-resolution displays, and connectivity of modern mobile phones to offer thermal imaging capabilities at a fraction of the cost of traditional professional systems [8]. This technological leap has opened unprecedented new avenues for point-of-care diagnostics and remote patient monitoring, holding particular promise for deployment in low- and middle-income countries (LMICs) and other low-resource settings where access to expensive medical equipment is limited. This class of smartphone-attachable thermographic devices comprises models from FLIR Systems such as One Pro, One Pro LT, E60bx, C2, B200 and SC305 [9,10,11], as well as alternatives from SEEK like Compact Pro and Thermal Compact XR. It is noted that SEEK devices are generally used for building diagnostics, outdoors, firefighting and commercial trades, making them less preferable for biomedical applications than FLIR devices [12].

Despite the rapid proliferation and adoption of smartphone-based thermal cameras in research and clinical exploration, the empirical evidence supporting their validity and reliability remains fragmented and often contradictory. A comprehensive review of the existing literature reveals a landscape of conflicting findings, which complicates efforts to establish clear guidelines for their clinical use [6,7,13,14,15,16].

On the ond hand, several studies have reported promising results, suggesting that these low-cost devices are reliable and can produce data comparable to that from high-end, professional-grade thermal cameras for certain specific applications [12,17,18]. For instance, research in diabetic foot assessment has indicated that smartphone thermography can effectively identify temperature asymmetries indicative of inflammation or poor perfusion, performing similarly to more expensive systems [11,19]. Likewise, in the field of plastic and reconstructive surgery, these devices have been successfully used for intraoperative perforator mapping and postoperative flap monitoring, where the primary goal is to visualize relative temperature differences that correlate with blood flow [14]. These studies highlight the potential of the technology for tasks dependent on identifying thermal patterns and relative temperature differentials within a single field of view [20]. On the other hand, a growing body of evidence raises significant concerns about the accuracy and agreement of these devices when used for measuring absolute temperatures. Studies comparing smartphone cameras to higher-end thermographic systems or gold-standard thermometers have frequently revealed significant discrepancies, systematic biases, and poor overall agreement [10,15].

This divergence in the literature points to a critical nuance that is often overlooked: the distinction between a device’s utility for assessing relative temperature patterns versus its ability to measure absolute temperature with clinical-grade accuracy. The validity of a device for one application (e.g., identifying a “hot spot”) cannot be automatically extrapolated to another (e.g., diagnosing a fever based on a specific threshold). This creates an “application-specific validity trap,” of sorts, where positive results from qualitative or relative assessments may foster a false sense of confidence in the device’s quantitative, absolute measurement capabilities. A primary contribution of the present study is to directly address this ambiguity by rigorously evaluating the FLIR One Pro’s performance specifically as an absolute temperature measurement tool.

The central research gap addressed by this study lies at the intersection of technological accessibility and clinical need. While sophisticated error-correction models are under development, and while the FLIR One Pro has been compared against expensive, high-end thermal cameras, there remains a significant paucity of empirical data from a more pragmatic comparison: its performance against another ubiquitous, low-cost, single temperature measurement and readily available clinical instrument like a non-contact infrared thermometer. The reference instrument used in this study, the iHealth PT3, belongs to a category of non-contact infrared thermometers (NCITs). Though in many medical applications one is interested in temperature asymmetry, poor validity and reliability limit the potential of plug-in cameras, for instance, in the case of automatic temperature checks for COVID-19 or the assessment of the evolution of an inflammatory chronic disease such as rheumatoid arthritis [21].

Secondly, we study the effects of algorithmic calibration for reconciling differences between the commercial and reference devices, inspired by recent studies [22,23,24]. Specifically, we applied a suite of eight machine learning regressors to map FLIR readings onto iHealth PT3 values, including robust polynomial regression, Deming regression, isotonic regression, quantile gradient-boosted regression trees (GBRT), monotone LightGBM, spline + Huber, LOESS, and weighted splines. Evaluation was performed using GroupKFold cross-validation by participant to prevent data leakage, and metrics included mean absolute error (MAE), root mean squared error (RMSE), bias, and Bland–Altman limits of agreement.

This paper is organized as follows. Section 2 presents the materials and methods. Section 3 presents the experimental results. Section 4 discusses the findings of this work. Section 5 concludes this work and recommends future directions.

## 2. Materials and Methods

**Figure 1:**
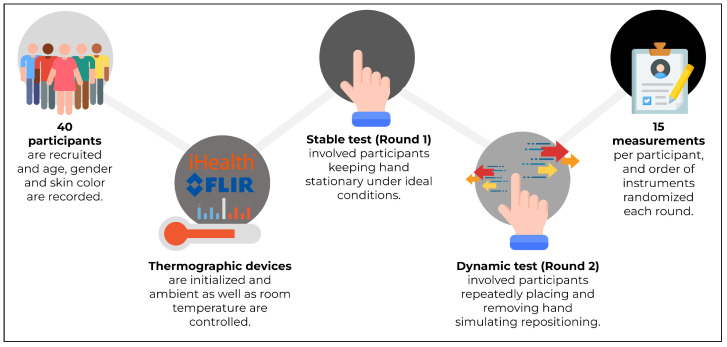
Overview of the study design described in the methodology for assessing the validity and reliability of a mainstream plug-in thermal camera (FLIR One Pro) for measuring skin temperature in comparison to a traditional standalone infrared thermometer (iHealth PT3). Participants placed their hands in custom 3D-printed stations to ensure consistent measurement precision in each round.

Each of the 40 participants completed two rounds of measurements. In Round 1, they remained stationary in a stable environment while their hand temperature was measured ten times with the iHealth PT3 (iHealth Lab Inc., Sunnyvale, CA, USA) and ten times with the FLIR One Pro (FLIR Systems, Wilsonville, OR, USA). The order of instruments was randomised. In Round 2, participants moved their hand slightly between measurements to introduce a dynamic element; again, ten measurements per device were recorded. After data collection, descriptive statistics, Bland–Altman agreement analyses, and reliability measures were computed for each round. Following this, machine learning based calibration methods were applied. This is summarized in Figure 1.

### 2.1. Study Objective

This investigation was designed as a method comparison and reliability study, conducted in accordance with the Guidelines for Reporting Reliability and Agreement Studies (GRRAS). The study protocol, including all procedures involving human participants, received full approval from the Institutional Review Board of the Mohammed Bin Rashid University of Medicine and Health Sciences in the United Arab Emirates. Prior to participation, all individuals were provided with a detailed explanation of the study procedures and objectives, after which they provided written informed consent to participate.

### 2.2. Study Design

Our research design followed the guidelines for conducting research with thermal cameras as suggested by Ring and Ammer [25] and included the following variables.

Participants: A cohort of 40 healthy adult participants was recruited for this study through convenience sampling from the faculty and staff members of the university. The sole inclusion criterion was an age of 18 years or older. No other specific exclusion criteria were applied, and a summary of their characteristics is in Table 1. This approach was justified on two grounds: first, the study’s primary endpoint was the comparison of measurement differences between the two instruments withineach participant, which minimizes the confounding effects of inter-individual physiological variability. Second, extensive research has established that infrared thermographic measurements of skin emissivity are not significantly affected by skin color or phototype, making it unnecessary to control for this variable in a method-comparison design [26,27,28]. As such, participant diversity does not confound measurement validity in this case; rather, it enhances external generalizability by ensuring robustness across typical variations in human skin.

Environment: This study took place within a controlled environment situated at a medical and health science university in the United Arab Emirates, and the dedicated room had dimensions of 5 m × 5 m × 3 m. The room temperature was set at 23 °C and controlled using air conditioning and controlled by a room thermometer. This specific temperature was selected as it falls within the range of accepted temperature, ranging from 18 °C to 25 °C [29]. This reduces the likelihood of subjects shivering at relatively low temperatures, and sweating at relatively high temperatures, with allowance for individual variations. This temperature is maintained and accounts for the heat generated by electronic equipment, and the maximum number of patients and staff present in the room. To avoid direct drafts that could affect skin temperature, the experimental site was positioned at least 2.5 m away from any airflow source. The room was illuminated with a stable LED lighting system, and the measurement station was shielded from any direct view of windows to prevent interference from external thermal radiation. Relative humidity was stable (40–50%), within the recommended range for thermographic imaging [4].

Measurement Instruments: Two commercially available, low-cost devices were used for temperature measurement. The characteristics of the two measurement instruments are presented in Table 2.

Test Instrument: The test instrument was the FLIR One Pro, a long-wave infrared (LWIR) thermal imaging camera attachment [30]. It was connected to an Apple iPhone 6S for operation and data visualization via the official FLIR ONE application. Temperature readings were obtained using the application’s integrated spot meter function.

Reference Instrument: The reference instrument was the iHealth PT3 Non-Contact Forehead Thermometer [31]. This is a consumer-grade medical device designed specifically for measuring human body temperature via infrared detection from the forehead. It provides a single-point temperature reading on a digital display.

Participant and Equipment Preparation: Upon entering the site, each participant was requested to wait at least 5 min for the skin to become acclimatized to the room temperature, for pre-imaging equilibrium [32]. This waiting time is similar to studies using the same type of camera [12,33]. A. During this period, demographic information, including age range and skin color (assessed using the six-point Fitzpatrick scale), was collected [34]. The FLIR One Pro thermal camera was switched on at least 20 min prior to the start of the first measurement to allow the device’s internal sensor and optics to stabilize and adapt to the room temperature, as recommended by best practices [9].

Measurement Procedure: To ensure consistency in measurement distance and hand positioning, two custom 3D-printed stations were fabricated. The field of view was selected to be 20 cm × 20 cm, as this is recommended for a single hand [25]. These stations standardized the distance between the subject’s hand and each measurement device according to their respective optimal specifications (15 cm for the FLIR One Pro, <3 cm for the iHealth PT3) and minimized participant movement during data acquisition. Temperature measurements were taken from the dorsal surface of each participant’s right hand. THE hand dorsum temperature varies slowly, so thermal recovery of the measurement site should remain consistent.

Experimental Rounds: The experiment consisted of two distinct rounds, each designed to collect 15 paired measurements from both instruments over a period of 150 s. The order in which each participant used the instruments was randomized at the start of each round to mitigate any potential order effects.

1. Round 1 (R1—Stable Environment): R1 was designed to assess the baseline repeatability and precision of each instrument under ideal, static conditions. After selecting the first instrument, participants placed their hand in the corresponding station and kept it stationary. A temperature measurement was recorded every 10 s for a total of 15 readings. The process was then immediately repeated with the second instrument.
2. Round 2 (R2—Dynamic Environment): R2 was designed to test instrument robustness and reliability in a dynamic scenario that mimics clinical repositioning or minor patient movement. For this round, participants were instructed to place their hand in the station for 5 s (the time required to take a reading), after which they would remove and then immediately replace their hand in the station for the next measurement. This cycle was repeated for all 15 readings with each instrument.

### 2.3. Statistical Analysis

All statistical analyses were performed using Python (3.10) [35] with Numpy (2.3.2) [36], Pandas, Scipy (1.16.1) [37] and Pingouin 0.5.5 [38] libraries.

The normality of the data distributions for the temperature differences was first confirmed using the Shapiro–Wilk test [39]. The alpha level for determining statistical significance was set a priori at p<0.05. Results showed values above 0.05, indicating that the data distributions followed a normal curve (R1 data *p*-value = 0.35; R2 data *p*-value = 0.26).

Recision Analysis: Descriptive statistics, including the mean and standard deviation (SD), were calculated for the 15 repeated measurements for each participant, instrument, and round. The intra-participant SD served as the primary metric for assessing the precision and repeatability of each device.

Mean Difference Analysis: Paired-samples *t*-tests [40] were conducted to determine if a statistically significant mean difference existed between the skin temperature estimates obtained from the FLIR One Pro and the iHealth PT3 within each experimental round (R1 and R2).

Agreement Analysis: The level of agreement between the two instruments was assessed using the Bland–Altman method [41]. This involved calculating the mean difference between the paired measurements (the bias) and the 95% limits of agreement (LoA). The LoA were calculated as the mean difference ± 1.96× SD of the differences, providing a range within which 95% of the differences between the two devices are expected to fall.

Correlation and Reliability Analysis: The linear relationship between the measurements from the two devices was evaluated using the Pearson correlation test for both R1 and R2. To assess reliability, the Intraclass Correlation Coefficient (ICC) [42] was calculated for absolute agreement between the devices for each round.

### 2.4. Machine Learning Regressors

The primary objective of this analysis was to develop and validate a regression model, *f*, that maps the raw temperature readings from the test instrument (FLIR One Pro, $t_{\mathrm{flir}}$) to the corresponding readings from the reference instrument (iHealth PT3, $t_{\mathrm{ihealth}}$), such that $t_{\mathrm{ihealth}}\approx f\left(t_{\mathrm{flir}}\right)$. The goal was to select the optimal model *f* that minimizes the prediction error and improves the clinical agreement of the corrected FLIR One Pro readings. For model development, the raw FLIR One Pro temperature served as the independent variable (feature, *X*), and the iHealth PT3 temperature served as the dependent variable (target, *Y*).

A diverse set of ten regression models was selected to explore different assumptions about the nature of the error between the two instruments. These regression models can be categorized into three methodological families: (1) Robust Statistical Baselines, (2) Non-parametric Smoothers, and (3) Tree-based Machine Learning Ensembles.

This selection criterion was driven by the need to address three specific characteristics of thermal measurement error observed in prior comparisons: the presence of outliers requiring robust loss functions (e.g., Huber loss), the physical necessity of a monotonic relationship (higher FLIR readings must correspond to higher reference readings), and the need to model complex, non-linear bias.

Robust Statistical Baselines: These models serve as interpretable benchmarks that account for measurement error and outliers without excessive complexity:

1. Polynomial regression (PolynomialFeatures + HuberRegressor), which balances sensitivity to nonlinear trends with resistance to outliers through Huber’s loss function [43].
2. Deming regression, a total least squares method that accounts for error in both predictor and response; the error variance ratio (λ) was estimated from within-participant replicate variances [44].

Non-parametric Smoothers: These methods allow for flexible functional forms that are not constrained by a specific equation, adapting to local variations in the data structure:

1. Isotonic regression, a nonparametric, order-constrained regression that enforces monotonicity and prevents non-physical downward mappings [45].
2. Spline + Huber, combining natural cubic splines with quantile-spaced knots and a robust Huber loss, allowing smooth nonlinear fits while limiting the influence of outliers [46].
3. LOESS (locally weighted scatterplot smoothing), which captures flexible nonlinear trends by fitting local polynomials with robustness weights [47].
4. Weighted splines, where spline regression was fit with inverse-variance weights derived from a pilot isotonic model to address heteroscedasticity in the error structure [48].

Tree-based Machine Learning Ensembles: To capture complex interactions and provide probabilistic outputs or variance reduction, we employed state-of-the-art ensemble methods:

1. Quantile gradient-boosted regression trees (GBRT), which extend boosting to conditional quantiles, estimating predictive distributions (2.5%, 50%, 97.5%) rather than just the mean [49].
2. LightGBM with monotone constraint, an efficient gradient boosting framework where the median predictor is fit under monotonicity constraints, with unconstrained quantile models for uncertainty intervals [50].
3. Random Forest, a bagging ensemble method that aggregates multiple decision trees to reduce variance and mitigate overfitting, particularly useful given the high noise observed in the thermal data [51].
4. Extra Trees (Extremely Randomized Trees), a variation of Random Forest that introduces further randomness in the split selection, often yielding lower variance and smoother decision boundaries than standard forests [52].

## 3. Results

As mentioned previously, a total of 40 participants were successfully enrolled and completed the study protocol. This resulted in the collection of 2400 individual temperature measurements (40 participants × 2 instruments × 2 rounds × 15 repeated measurements per round). Approximately two-thirds of our convenience sample consisted of females (*n* = 27), who tended to be younger and have a darker skin phototype compared to the male participants (*n* = 13).

For the calibration step, to avoid leakage, performance was estimated with GroupKFold cross-validation by participants (5 folds where possible). We report the Mean Absolute Error (MAE), Root Mean Squared Error (RMSE), Coefficient of Determination ($R^{2}$), bias (mean error), and 95% Bland–Altman LoA from cross-validated predictions. For deployment, models were fit on the full dataset; 95% prediction intervals were obtained from (a) the quantile models directly or (b) a conformal absolute-residual quantile for point estimators.

All data were recorded and subsequently entered into a digital spreadsheet for analysis. For the FLIR One Pro, the temperature was read directly from the spot meter function of the mobile application, which was aimed at the center of the thermal image of the hand dorsum. For the iHealth PT3, the single numerical value displayed on the device’s screen was recorded.

### 3.1. Instrument Precision and Repeatability

**Figure 2:**
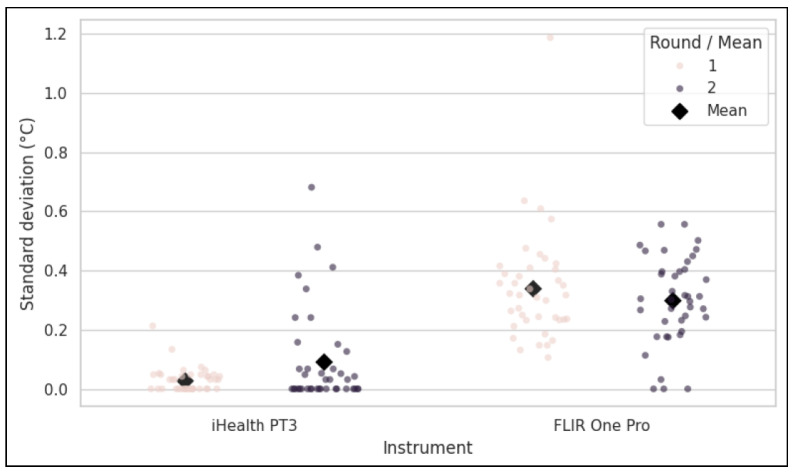
Standard deviation of the measurement instruments within each round.

The precision of each instrument was evaluated by calculating the intra-participant standard deviation (SD) of the 15 repeated measurements in each round, as shown in Figure 2. In the stable environment (R1), the reference instrument (iHealth PT3) demonstrated high precision, with a mean intra-participant SD of 0.030 °C. In contrast, the test instrument (FLIR One Pro) showed substantially lower precision, with a mean intra-participant SD of 0.340 °C, representing more than a ten-fold increase in measurement variability under ideal conditions.

This pattern of disparate precision persisted in the dynamic environment (R2). The iHealth PT3’s precision was slightly lower with a mean SD of 0.093°, while the FLIR One Pro again exhibited high variability, with a mean SD of 0.300 °C. These results indicate that the FLIR One Pro possesses significantly higher intrinsic measurement noise compared to the iHealth PT3, a characteristic that was not mitigated by the experimental condition.

**Figure 3:**
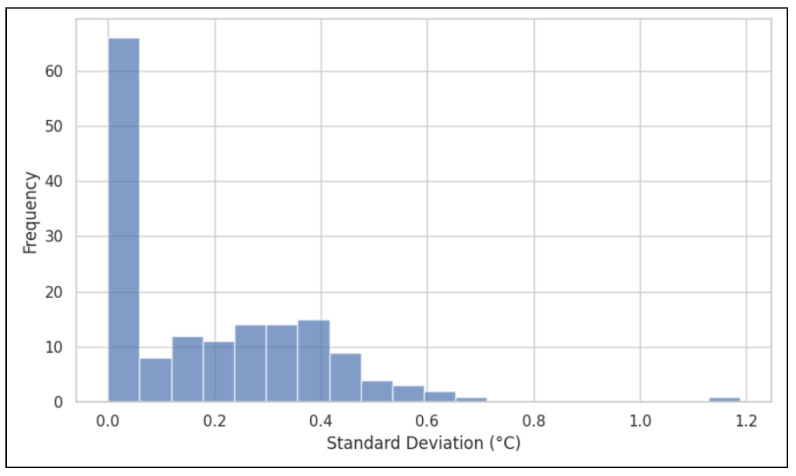
Histogram distribution of intra-participant standard deviations.

As observed in Figure 3, the distribution of precision across all instruments and rounds highlights how tightly clustered iHealth PT3 SDs are compared to FLIR One Pro SDs.

### 3.2. Comparison of Mean Temperature Readings and Correlation

A one-sample *t*-test on the measurement bias confirmed that the observed differences between the instruments were highly statistically significant in both the stable environment (R1: t-statistic = −18.46, *p*-value $<10^{-37}$) and the dynamic environment (R2: t-statistic = −14.38, *p*-value $<10^{-37}$). The infinitesimally small *p*-values lead to an unequivocal rejection of the null hypothesis—that there is no difference between the devices—in both experimental conditions.

**Figure 4:**
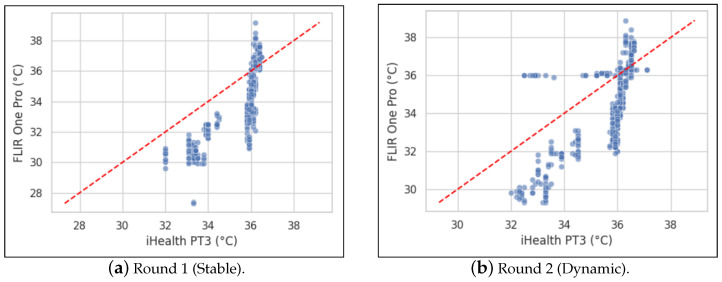
Pearson correlation between instruments across measurement rounds.

Further analysis explored the Pearson correlation between the devices as shown in Figure 4. When comparing paired measurements within each round, a moderate positive correlation was found (R1: r ≈ 0.79; R2: r ≈ 0.76). This indicates that, within a stable or dynamic session, the absolute temperatures recorded by the devices tend to rise and fall together. However, a separate analysis was conducted to assess how each instrument responded to the change in environment between rounds. For each participant, the change in mean temperature from R1 to R2 was calculated for each device, and these changes were correlated. This resulted in a near-zero correlation (r ≈ −0.04, *p* ≈ 0.80), confirming that the way each instrument responds to environmental change is unrelated. This clarifies that while absolute temperatures show some correlation, the devices’ responses to dynamic shifts are not consistent with each other.

### 3.3. Agreement and Reliability Between Instruments

The clinical agreement between the two instruments was assessed using Bland–Altman analysis, which revealed a clinically unacceptable level of disagreement in both experimental rounds. Our mean bias is defined during computation as reference–test instrument.

In the stable environment (R1), the analysis showed a mean bias of −1.42 °C, indicating that, on average, the FLIR One Pro systematically recorded temperatures 1.42 °C lower than the iHealth PT3. More critically, the 95% LoA were exceptionally wide, ranging from −4.44 °C to +1.60 °C. This signifies that for any given measurement, the reading from the FLIR One Pro could be expected to be as much as 4.44 °C lower or 1.60 °C higher than the reading from the reference thermometer, with 95% confidence. The total range spanned by the LoA was 6.03 °C. The LoA span exceeds both the manufacturer’s stated accuracy (±3 °C) and typical clinical tolerance thresholds (±0.3–0.5 °C) used in fever screening [15]. This means the FLIR One Pro’s random error is an order of magnitude greater than what is acceptable for diagnostic use. Practically, such variability could lead to false negatives or positives in infection detection or febrile screening applications, thereby rendering the device unsuitable for absolute temperature assessment without calibration.

In the dynamic environment (R2), the results were similarly poor. The mean bias was −1.15 °C, with 95% LoA extending from −4.29 °C to +1.99 °C, for a total range of 6.28 °C. The ICC results showed moderate reliability in both the stable environment (ICC ≈ 0.50) and the dynamic environment (ICC ≈ 0.55), strengthening the conclusion that the devices do not agree sufficiently for clinical substitution. A comprehensive summary of these quantitative findings is presented in Table 3.

**Figure 5:**
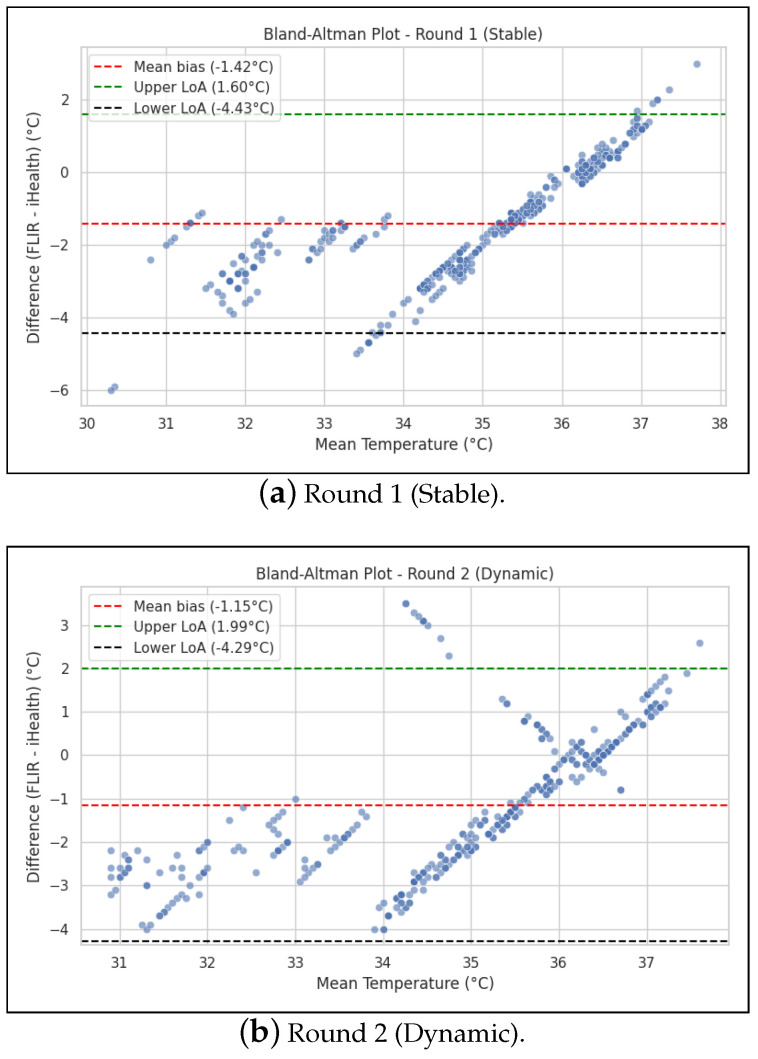
Bland–Altmananalysis for correlation between instruments across measurement rounds.

Bland–Altman analysis demonstrated wide variability between the two measurement instruments for both rounds (Figure 5). In R1, the mean bias was 1.35 °C with a lower limit of −1.56 and an upper limit of 4.25. One can observe positive differences for lower temperatures and negative differences for higher temperatures. This pattern is similar in R2, which showed a mean bias of 1.38 °C with a lower limit of −1.36 and an upper limit of 4.11. With these values, the precision of the thermal camera FLIR One Pro exceeded the manufacturing values, i.e., ±3 °C. Furthermore, three measurements fell outside the limits of agreement, two being from the same participant (i.e., lower limit in R1 and R2).

Within each round, the Pearson correlation between iHealth PT3 and FLIR One Pro measurements was moderate (r ≈ 0.79 in the stable environment and r ≈ 0.76 in the dynamic environment), indicating that the two devices track temperature changes reasonably well. However, when correlating the changes between rounds, the correlation was essentially zero (r ≈ −0.04, *p* ≈ 0.80), confirming our assertion that the instruments’ responses to environmental changes are unrelated.

### 3.4. Calibration Performance

**Figure 6:**
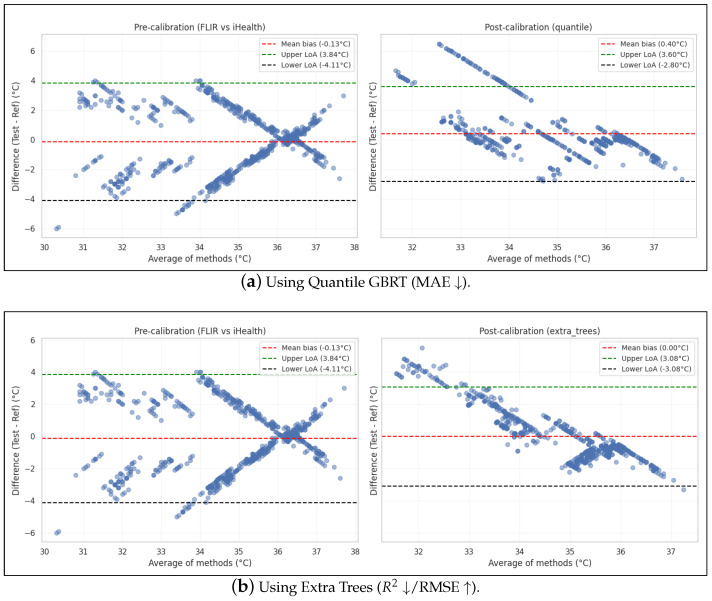
Bland–Altman comparison of FLIR vs. iHealth before and after calibration with the best performing models (across different metrics).

Table 4 compares ten calibration strategies. As shown in Figure 6, the raw FLIR One Pro minus iHealth PT3 differences exhibit temperature-dependent bias (X-shaped pattern) on the left. This suggests that the camera over-reads at low temps and under-reads at high temps. Post-calibration differences (left) using the best MAE model (Quantile GBRT) and best RMSE/$R^{2}$ model (Extra Trees) show reduced bias and slightly narrower limits of agreement, yet residual spread remains relatively large. This is indicated by the downward slope (which is common when the reference device itself has some noise), but the data is relatively more linear and contained. We report the hyperparameter ranges considered in Table 5.

Across the paired observations from participants, all models reduced systematic bias relative to raw FLIR values but left substantial random error. The lowest MAE was obtained with quantile GBRT (MAE = 1.162 °C, RMSE = 1.880 °C, $R^{2}$ = 0.065). Notably, while GBRT minimized absolute error, the Extra Trees regressor achieved the lowest RMSE (1.792 °C) and the highest explained variance ($R^{2}$ = 0.152), effectively eliminating systematic bias (bias ≈ 0 °C). Other models that effectively reduced systematic bias included Monotone LightGBM (bias +0.028 °C), Isotonic (bias +0.033 °C), and Deming (bias +0.035 °C). Post-calibration Bland–Altman limits for the best MAE model (Quantile GBRT) narrowed to −2.80 to +3.60 °C, while the best RMSE model (Extra Trees) showed symmetric limits of −3.08 to +3.08 °C. Despite these improvements over raw values (limits of approx. ±4.0 °C), the persistent wide intervals indicate that measurement variance dominates residual error even after bias correction. Thereby, it appears that calibration can account for accuracy (bias) but cannot fix the precision (sensor noise) in such applications as per our empirical results.

## 4. Discussion

### 4.1. Principal Findings

Smartphone plug-in cameras can be acquired for a fraction of the price of professional thermal cameras and offer new use cases due to their high usability. Among these use cases, medicine has drawn the attention of many researchers who have demonstrated the capacity of the smartphone plug-in thermal cameras for medical diagnosis and monitoring of treatments [10,12,53]. The fundamental advantage of a thermal imaging camera over a single-point infrared thermometer is its ability to provide spatial context. A single-point device, such as the iHealth PT3, provides a single, non-contextualized data point—a single pixel of information from a complex physiological landscape. In contrast, a thermal camera generates a thermogram, or heat map, which is a two-dimensional visualization of the thermal distribution across an entire surface. In many clinical scenarios, diagnostic and monitoring decisions are based not on a single absolute temperature but on the analysis of thermal gradients, patterns, and asymmetries, which are only visible on a heat map [4,54,55].

Though some studies investigated the accuracy of low-cost thermal cameras and the FLIR One in particular [9], to date, no research has investigated the bias and repeatability of such camera on human skin, which was the aim of this paper.

As per our results, the combination of a significant systematic bias (systematically under-reading by over 1 °C) and exceptionally wide limits of agreement—spanning a range of over 6 °C—demonstrates that the uncalibrated output from this device is not interchangeable with that of a dedicated clinical thermometer. The high intrinsic variability (i.e., low precision) of the FLIR One Pro, which was more than ten times greater than that of the iHealth PT3 in stable conditions, and the moderate ICC (≈0.50–0.55) further compound its limitations, severely constraining its utility for applications that require reliable and repeatable measurements of absolute temperature. This lower precision is to be expected, as relatively cheaper thermal cameras use uncooled microbolometer sensors, which have more intrinsic noise compared to the cooled detectors found in costlier variants [56]. Thus, the intrinsic variability most likely stems from the hardware design limitations of uncooled microbolometer arrays (typically VOx detectors). These sensors exhibit thermal drift, non-uniform response, and temperature-dependent noise due to the absence of active cooling [57]. Additional factors include the 160 × 120 pixel resolution, fixed-focus optics, and lack of in-field lens calibration, all of which degrade radiometric precision over time [8]. The combination of low spatial resolution and high temporal noise explains the higher intra-participant SD (0.30–0.34 °C) observed in this study.

In an effort to assess whether post hoc calibration could mitigate these deficiencies, we evaluated a diverse suite of ten machine learning regressors, extending from robust statistical baselines to tree-based ensembles including Random Forest and Extra Trees. These models highlighted a persistent bias–variance trade-off: while methods such as Deming, isotonic, Monotone LightGBM, and Extra Trees effectively eliminated mean bias (to <0.06 °C), they could not sufficiently suppress random error. Although the Extra Trees regressor achieved the best variance reduction ($R^{2}$ = 0.152, RMSE = 1.792 °C), the performance plateau across all nonlinear models (MAE ≈ 1.16–1.49 °C) underscores that model complexity cannot compensate for intrinsic measurement noise arising from factors such as sensor resolution, distance, and emissivity. Clinically, this means that even with advanced calibration, corrected FLIR readings fall far short of the fever-screening thresholds (≤0.3–0.5 °C MAE) recommended in regulatory guidance. Consequently, the primary value of these models lies not in correction, but in quantifying uncertainty; quantile approaches and prediction intervals make explicit the substantial unreliability inherent in the hardware.

To be fair, despite being used in clinical settings, the FLIR One is not commercialized as a medical measurement device nor marked as such. A critical aspect of interpreting these findings involves comparing the device’s observed performance with its own manufacturer-stated specifications (±3 °C or ±5% of the reading, whichever value is greater). This severely hinders the device’s ability to respond to phenomenon such as inflammation [58] or rheumatoid arthritis [59] that have skin temperatures not exceeding 2 °C. In a particular case of children suffering from rheumatoid arthritis, the difference in temperature does not vary more than 0.6 °C [60]. The mean intra-participant standard deviation of the FLIR One Pro found in our study (0.34 °C) is already more than half of this clinically meaningful threshold. When considering the much larger potential error indicated by the limits of agreement, it becomes clear that the device’s intrinsic measurement noise would completely obscure the subtle thermal signals associated with changes in disease activity.

This highlights a crucial distinction between the concepts of “accuracy” as often presented in technical specifications and “agreement” as assessed for clinical utility. A manufacturer’s accuracy is typically determined under idealized laboratory conditions, often against a stable, uniform blackbody calibration source. It provides a single, often optimistic, value representing the maximum expected error. Clinical agreement, as quantified by the Bland–Altman method, provides a far more realistic and meaningful assessment of performance in a real-world application. It deconstructs error into its systematic (bias) and random (precision) components, revealing not just how much a device might be wrong, but in what direction it tends to be wrong and how unpredictably it varies around that tendency. The results of this study strongly suggest that for clinical decision-making, an evaluation of agreement is superior to a simple reliance on manufacturer-stated accuracy, as the latter can be dangerously misleading by masking the true extent of a device’s measurement uncertainty.

### 4.2. Comparison with Related Work

In another publication, when used to measure lower limb temperature, the FLIR One has been reported to overestimate the temperatures (up to 3 °C) compared to a high-end thermal camera (i.e., FLIR E60bx) [10]. At higher temperatures (e.g., 30–35 °C), the trend has been reversed, with temperatures up to −2 °C lower than the FLIR E60bx. Our results followed a similar pattern despite using a more advanced generation of camera that has a thermal resolution two times higher (160 × 120 compared to 80 × 60).

We similarly observed the overestimation of skin temperature in the low-temperature range (29.9–35.4 °C as displayed by the FLIR One Pro, differences of 0.81 to 4.39 °C compared to the iHealth) and underestimation with higher temperatures (36.1–38.2 °C as displayed by the FLIR One Pro, differences of −1.92–0.03).

Though the temperature differences in this research are higher compared to Machado et al. [10], it is worth noting that in the lower limbs study, the FLIR One was compared with another thermal camera from the same brand that might also follow the same pattern of over- and underestimation. In another study, Vardasca et al. [9] identified overestimation of temperature values when taking measurements between 20 and 40 °C of a certified and calibrated blackbody plate with the FLIR One. Readings of the FLIR One showed temperatures always exceeding the set temperatures up to 2.7 °C. In the same research, the authors identified differences up to 1.6 °C between iOS and Android phones. Their study identified that when plugged into an iOS device, the FLIR One camera readings are higher at lower temperatures (20–30 °C) and lower at higher temperatures (30–40 °C) compared to the same camera plugged into an Android phone.

Previous studies have reported similar limitations across low-cost smartphone thermography systems: with one study [11] validating the SEEK Compact Pro for diabetic-foot screening, showing accuracy within ±1 °C only after calibration, another [12] finding FLIR and SEEK devices comparable for relative temperature mapping but inconsistent for absolute readings. Thus, the FLIR One Pro’s performance aligns with the broader trend: reliable for qualitative thermal pattern analysis but unreliable for quantitative thermometry without correction models.

In comparison with our study, measurements obtained with the blackbody calibration source at the same temperatures show lower biases (assuming the accuracy of the iHealth instrument) but higher standard deviation. These differences could be explained by the homogeneous temperature of the blackbody plate as opposed to the hand that showed temperature differences of up of 5 °C between the extremities and the top of the hand. These higher differences could potentially influence temperature readings. More generally, differences in temperature with the FLIR One Pro could also be explained by the limitation of the camera to adapt to high emissivity; while the maximum emissivity sensibility of the camera for “matte” surfaces is 0.95, the blackbody plate and human skin have respective emissivities of 1.0 and 0.97–0.99 [3]. This could likely contribute to the systematic under-reading of temperature as well.

While unsuitable as a standalone thermometer, the FLIR One Pro retains value for pattern-based or relative thermography—for instance: detecting asymmetrical heat patterns in vascular or inflammatory assessments, intraoperative perfusion monitoring or post-operative flap observation where relative differences are diagnostically meaningful [7,14]. Therefore, its utility lies in spatially comparative rather than absolute quantitative applications. This distinction underscores the device’s potential role as an accessible screening or educational tool, pending future integration of AI-based calibration algorithms [23,61].

### 4.3. Limitations

Though the instruments used in this research are not commonly compared, thus offering new information to the research community, it is limited by two considerations.

Firstly, while this study provides an “apples-to-apples” comparison between two widely available, consumer-grade infrared devices under identical conditions, neither instrument was calibrated against a gold-standard reference such as a certified blackbody source. Prior studies have shown that lack of calibration in portable thermometers and low-cost thermal cameras can introduce systematic bias [9,15,61]. Thus, our findings primarily reflect the relative performance of these devices, rather than their absolute validity for core body temperature estimation. Secondly, this study is constrained by the anatomical disparity between surface temperature sites and true core body temperature, particularly given that the reference thermometer is designed for forehead use. It has been observed that there are slight differences between forehead and wrist-based measurements [62,63].

Although this research sought a heterogeneous data sample and diversity among participants, it does not allow us to statistically draw conclusions when it comes to correlation between age group, gender, skin color, and temperature variability. For this reason, we recommend future research to include a calibration phase for the measurement instruments as well as a more homogeneous data sample to compare research outcomes. However, a one-way ANOVA confirmed that the measurement bias was not significantly affected by participants’ skin color. The F-statistic (≈0.48) and high *p*-value (≈0.75) indicate no systematic difference in bias across skin-colour categories, suggesting that it is not a primary driver of the observed disagreement.

## 5. Conclusions

This study provides a rigorous quantitative comparison of a smartphone-based thermal camera and a non-contact infrared thermometer for the measurement of absolute skin temperature. The findings demonstrate unequivocally that the FLIR One Pro, when used without specific calibration, exhibits poor precision and a clinically unacceptable level of agreement with the reference thermometer. The magnitude of the systematic bias and the extremely wide range of random error far exceed both the manufacturer’s own accuracy specifications and any clinically tolerable margin of error for diagnostic or monitoring purposes.

The primary contribution of this research is the clear, evidence-based conclusion that the uncalibrated FLIR One Pro should not be used as a substitute for a dedicated clinical thermometer in any application where precise and reliable absolute temperature values are required. While the allure of its low cost, portability, and novel technology is strong, its raw performance is insufficient for tasks such as fever screening, infection detection, or the longitudinal monitoring of inflammatory conditions.

In this study, a comprehensive suite of calibration models ranging from robust baselines to advanced tree-based ensembles successfully reduced the systematic bias between FLIR and iHealth measurements. While Quantile GBRT achieved the lowest MAE (1.162 °C), the Extra Trees regressor provided the best overall fit, improving explained variance to $R^{2}$ = 0.152 and narrowing Bland–Altman limits to approximately ±3.1 °C. However, even with these state-of-the-art ensemble methods, the explained variance remains low, and prediction intervals stay several degrees wide. These results indicate that under our acquisition setup, random error dominates and cannot be sufficiently corrected by function-fitting alone. For screening use, improvements must come from sensor/measurement design (distance control, emissivity, ambient compensation, multi-pixel aggregation) and additional features, not just algorithmic calibration.

Looking forward, the potential of low-cost thermal imaging in medicine is not entirely negated by these findings. Current evidence indicates that even without perfect absolute accuracy, these systems retain clinical utility for monitoring relative physiological trends. This is supported by recent scoping reviews highlighting their versatile clinical applications [64,65] despite implementation challenges [66], as well as specific validations in tracking perfusion gradients during burn wound rehabilitation [67]. Crucially, relative or pattern-based monitoring remains a viable and necessary alternative in resource-limited settings where high-end thermographic equipment is unaffordable.

To overcome the limitations in absolute measurement, future systems could learn to compensate for inherent hardware biases through advanced software. Recent research demonstrates that deep learning-driven correction approaches can reduce measurement variance by half, enabling accurate disease classification in diabetic foot screening where raw absolute temperatures fail [68]. Furthermore, the successful deployment of complex pipelines integrating semantic segmentation and non-rigid registration [68] directly onto mobile platforms confirms that these data-driven decision support tools are computationally feasible for point-of-care use [5]. However, until such rigorous AI-based compensations are embedded into consumer-grade applications, practitioners must exercise extreme caution and rely on validated medical devices for critical temperature measurements, particularly given the significant temporal instability and heating artifacts recently characterized in these compact modules [69]. Future work aimed at ensuring absolute accuracy should ideally combine these algorithmic advancements with robust physical calibration standards, such as the use of traceable blackbodies and multi-point calibration.

## Figures and Tables

**Table 1:** Characteristics of Participants.

Characteristic	Female (27)	Male (13)	Total (40)
Age range			
18–24	5	1	6
25–30	6	1	7
31–35	12	0	12
36–40	1	4	5
41–45	0	3	3
46–50	1	1	2
51–55	2	2	4
56–60	0	1	1
Skin color (Fitzpatrick scale)			
1	2	0	2
2	6	3	9
3	7	5	12
4	9	2	11
5	4	2	6
6	0	0	0

**Table 2:** Characteristics of the measurement instruments.

Feature	FLIR One Pro	iHealth PT3
Applications	Buildings, biomedicine, industry, lab tests	Medicine (skin temperature)
Detector type	Thermal and photon	Infrared
Output	160 × 160 radiometric jpeg	Temperature in °C or °F
Measurement ranges	−20 to 400 °C	32 to 42.9 °C
Focus/optimal distance	15 cm	3 cm
Emissivity	0.95	N/A
Resolution	One decimal	One decimal
Precision	±3 °C	±0.2 °C (within 35 °C to 42 °C range)

**Table 3:** Descriptive statistics and agreement analysis between the iHealth PT3 and FLIR One Pro across Round 1 (Stable) and Round 2 (Dynamic).

Parameter	Round 1 (Stable)	Round 2 (Dynamic)
Descriptive Statistics		
Mean Temperature (°C)	35.50	34.08
Mean Intra-participant SD (°C)	0.030	0.340
Agreement Analysis		
Mean Bias (°C)	−1.42	−1.15
Lower LoA (°C)	−4.44	−4.29
Upper LoA (°C)	+1.60	+1.99
LoA Range (°C)	6.03	6.28
ICC (2,1)	0.501	0.554
Pearson Correlation	0.788	0.760

**Table 4:** Cross-validated calibration performance (GroupKFold by participant, n=40). Metrics: mean absolute error (MAE), root mean squared error (RMSE), $R^{2}$, bias (mean error), and Bland–Altman LoA.

Model	MAE (°C)	RMSE (°C)	$R^{2}$	Bias (°C)	LoA (°C)
Polynomial (deg2)	1.414	1.909	0.037	+0.409	−3.25⋯+4.07
Deming	1.465	1.827	0.118	+0.035	−3.55⋯+3.62
Isotonic	1.442	1.842	0.103	+0.033	−3.58⋯+3.65
Spline + Huber	1.368	1.977	−0.033	+0.322	−3.50⋯+4.15
LOESS	1.265	1.857	0.089	+0.154	−3.47⋯+3.78
Weighted Spline	1.484	1.951	−0.006	+0.113	−3.71⋯+3.93
Quantile GBRT	1.162	1.880	0.065	+0.369	−3.25⋯+3.99
LGBM (monotone)	1.432	1.837	0.108	+0.028	−3.57⋯+3.63
Random Forest	1.278	1.845	0.101	+0.080	−3.53⋯+3.69
Extra Trees	1.369	1.792	0.152	+0.059	−3.45⋯+3.57

**Table 5:** Model hyperparameters, current values, and search ranges considered during tuning. Reported values reflect the final selected configuration after cross-validation.

Model	Hyperparameter	Current Value	Search Range
Polynomial (deg2)	Degree	2.0	[1, 2, 3]
	Alpha (regularization)	0.0	[0.0001, 0.001, 0.01]
	Epsilon (robustness)	1.35	[1.1, 1.35, 1.5, 1.7]
Deming	Lambda ratio	∼9.92	N/A
Spline + Huber	Knots	6	[3, 4, 5, 6, 8]
	Epsilon	1.35	[1.2, 1.35, 1.5]
LOESS	Frac (smoothing)	0.25	[0.1 …0.6]
	Iterations	1	[0, 1, 3]
Quantile GBRT	N estimators	400	[100, 300, 500]
	Learning rate	0.05	[0.01, 0.05, 0.1]
	Max depth	3	[2, 3]
LGBM (monotone)	N estimators	600	[100, 300, 500]
	Learning rate	0.03	[0.01, 0.05, 0.1]
	Num leaves	31	[7, 15, 31]
Random Forest	N estimators	400	[100, 300, 500]
	Min Samples Leaf	5	[1, 3, 5]
	Bootstrap	True	[True, False]
Extra Trees	N estimators	400	[100, 300, 500]
	Min Samples Leaf	5	[1, 3, 5]
	Bootstrap	False	[True, False]

## Data Availability

The original contributions presented in this study are included in the article/Supplementary Material. Further inquiries can be directed to the corresponding author.

## Supplementary Materials

The following supporting information can be downloaded at: https://www.mdpi.com/article/10.3390/s26041295/s1, Data and Code for Experiments.
